# Predictors of the use of interventions to prevent malaria in pregnancy in Cameroon

**DOI:** 10.1186/s12936-017-1786-z

**Published:** 2017-03-27

**Authors:** Jodie Dionne-Odom, Andrew O. Westfall, Tobias O. Apinjoh, Judith Anchang-Kimbi, Eric A. Achidi, Alan T. N. Tita

**Affiliations:** 1 Division of Infectious Diseases, Department of Medicine, University of Alabama at Birmingham, 908 20th Street South, Room 325A, Birmingham, AL 35294-2050 USA; 2 Department of Biostatistics, School of Public Health, University of Alabama at Birmingham, Birmingham, AL USA; 3 Department of Biochemistry and Molecular Biology, University of Buea, Buea, Cameroon; 4 Department of Zoology and Animal Physiology, University of Buea, Buea, Cameroon; 5 Division of Maternal-Fetal Medicine, Department of Obstetrics and Gynecology and Center for Women’s Reproductive Health, University of Alabama at Birmingham, Birmingham, AL USA

**Keywords:** Malaria in pregnancy, Malaria prevention, Sulfadoxine–pyrimethamine (SP), Bed nets, Cameroon

## Abstract

**Background:**

Malaria in pregnancy is common in sub-Saharan Africa where it contributes to perinatal morbidity and mortality. Use of insecticide-treated bed nets and intermittent preventive therapy with sulfadoxine–pyrimethamine during pregnancy are effective but underutilized interventions to prevent infection. Factors associated with bed net ownership and usage, and use of prophylaxis among recently pregnant women in Cameroon were investigated.

**Methods:**

National data from the 2011 Cameroon Demographic Health Survey was used to identify women with a pregnancy within the previous 5 years. Logistic regression models were created to assess for independent predictors of reported bed net ownership, bed net usage, and the use of malaria prophylaxis medications during pregnancy.

**Results:**

Nearly one in two women surveyed had a recent pregnancy (n = 7647). In this group, bed net ownership and usage rates were low (33.7 and 16.9%, respectively); 61.6% used medication for malaria prophylaxis during pregnancy. Bed net ownership and usage were associated with maternal literacy (aOR 1.4 for net usage, 95% CI 1.1–1.8) and the presence of children under age 5 in the home (aOR 2.3 for net usage, 95% CI 1.6–3.3). The use of malaria prophylaxis medication was associated with measures of healthcare access (aOR 17.8, 95% CI 13–24.5 for ≥4 antenatal care visits), higher maternal education (aOR 1.5, 95% CI 1.1–2.1) and maternal literacy (aOR 1.4, 95% CI 1.1–1.7).

**Conclusions:**

Women in Cameroon and their antenatal providers missed many opportunities to prevent malaria in pregnancy. Efforts toward ensuring universal bed net provision, consistent antenatal care and the education of girls are likely to improve birth outcomes attributable to malaria infection.

## Background

There were 214 million cases of malaria reported worldwide in 2015 and an estimated 438,000 associated deaths [1]. Pregnant women are particularly susceptible to malaria and the highest infection rates worldwide are in sub-Saharan Africa [2–4]. Prevention of malaria in pregnancy can reduce the risk of severe maternal anaemia by 38%, low infant birthweight by 43% and perinatal mortality by 27% [5]. Prevention is important since many pregnant women with malaria are asymptomatic. Currently available preventive interventions include insecticide-treated bed nets (ITNs) and malaria prophylaxis regimens (intermittent preventive therapy or IPT). The World Health Organization (WHO) cites universal access to malaria prevention tools as one of the pillars of the pathway toward malaria elimination [6].

Insecticide-treated bed nets are effective and one of few malaria prevention options that are recommended during the first trimester of pregnancy [7–12]. Current ITN ownership rates range from 3 to 80% and rates of ITN usage in pregnant women in sub-Saharan Africa are low (17% in one recent review) [1, 13, 14]. ITN ownership and usage have been associated with higher levels of education, income and urban residence [13]. There was a nationwide bed net campaign in Cameroon in 2011; 9 million nets were distributed at facility and community levels. Malaria prophylaxis with intermittent sulfadoxine–pyrimethamine (IPT-SP) during the 2nd and 3rd trimesters of pregnancy has been recommended by WHO in areas of moderate to high malaria transmission since 2004. IPT-SP is recommended for prophylaxis during pregnancy in Cameroon and has been available at no cost since 2006 [15]. SP has efficacy for 4–6 weeks after a single dose and improved outcomes are seen with repeated dosing [16–18]. Based on this data and concerns about emerging resistance, WHO recommends provision of at least three doses of SP in ANC clinic; Cameroon transitioned from a 2-dose to a 3-dose recommendation for IPT-SP in 2012 [19–21]. In order to improve malaria prevention in the vulnerable population of pregnant women, we sought a better understanding of the usage of available interventions. Data from a large national survey was used to identify factors associated with access to and use of malaria prophylaxis tools among women with a recent pregnancy in Cameroon where malaria is endemic.

## Methods

### Study design and population

Surveys were collected in Cameroon between January and August of 2011 as part of the cross-sectional National Demographic Health Survey (DHS) [22]. These recurring household level surveys have been carried out using previously described methods and survey data was weighted to make it nationally representative [22–24]. Data was collected from 15,426 eligible women who were interviewed using two-stage stratified sampling techniques. This study captures responses from the subset of 7647 women who reported a birth in the previous 5 years. Surveys included detailed questions about socio-demographics, pregnancy history, access to antenatal care services, site of delivery and the use of interventions to prevent malaria during the most recent pregnancy. Blood was collected from a subset of women for rapid HIV testing. DHS data has been cleaned, is devoid of personal identifiers and datasets are publicly available to researchers upon request.

### Definitions

#### Outcome variables

1. Bed net ownership. This DHS query was “Do you have a bed net at your house that can be used for sleeping?” The ownership of any type of bed net was captured.
2. Bed net usage. Among those who owned a bed net, women were asked the standard Roll Back Malaria Initiative indicator “Did you sleep under a bed net on the night prior to the survey?”
3. Usage of any malaria prophylaxis medication during the most recent pregnancy (not including the current pregnancy).
4. Number of doses of prophylactic sulfadoxine–pyrimethamine (SP) received (during the most recent pregnancy). This was categorized into 3 separate dichotomous variables: 0 vs 1+ doses; 0–1 vs 2+ doses; 0–2 vs 3+ doses.

#### Independent variables

Sociodemographics included age, partner’s age, education, literacy (ability to read a written phrase in the language of choice), partner’s education, marital status, polygamy, religion, urban/rural residence (rural defined as a population density <20,000 people), region of the country (10 official regions plus separate categories for the two largest urban areas; Douala and Yaoundé i.e., 12 regional categories), ownership of a means of transportation (bicycle, motorcycle or car), parity, number of children <5 years of age in the household, HIV status, current contraception use according to the WHO categories (modern = sterilization, pill, intrauterine device, injectable, implant, condoms or lactational amenorrhea; traditional = rhythm method or withdrawal), employment (in the past 12 months) and wealth. Wealth was categorized by DHS into quintiles (numbered 1–5 in order of increasing wealth) based on a composite measure of the household cumulative living standard [24]. Measures of health care utilization and access included visiting a health facility in the past 12 months, type of antenatal care provider seen at least once (physician, nurse or auxiliary nurse), location of antenatal care (hospital, health centre or home), number of total antenatal clinic visits, timing (trimester) of the first antenatal clinic visit and the location of delivery.

### Statistical analysis

Descriptive statistics were used for women surveyed who reported a pregnancy in the previous 5 years. Logistic regression models were fit to evaluate associations between independent variables and the six dichotomous outcomes of interest (bed net ownership, bed net usage, use of any medication for malaria prophylaxis, and 3 separate SP dosing measures (at least 1 vs >1 dose, at least 2 vs >2 doses, at least 3 vs >3 doses). Separate univariate (UV) and multi-variable (MV) models were fit for each outcome. All variables in Table 1 were considered for the MV models and variables were selected based on statistical significance in the univariate models (p < 0.05), review of existing literature and collinearity considerations. The same set of independent variables was used in all MV models. Sensitivity analyses were performed by fitting various MV models with and without the variables that showed collinearity. Since region of residence and urban/rural residence were highly collinear with the wealth variable, they were excluded from the MV models. The timing of the initial ANC visit, provider type and facility type were also excluded from MV models due to collinearity with one another. Missing data is presented in the tables but data points were generally complete. UV and MV odds ratios with 95% confidence intervals are presented in the tables and UV and MV odds ratios are shown in a figure. All analyses were performed with SAS 9.4 (Cary, NC) and results were adjusted for weighting, clustering and stratification using the SAS/STAT^®^ “SURVEY” procedures.

**Table 1:** Characteristics of women with pregnancy in the past 5 years (n = 7647)

Characteristic	N (%)
Demographics	
Age-median (IQR)	26.6 (22.0–32.4)
Age of husband/partner-median (IQR) (n = 6417)^a^	35.9 (29.7–43.4)
Relationship status	
Married	5248 (68.6)
Living with partner	1286 (16.8)
Widowed, divorced, or separated	482 (6.3)
Never in union	631 (8.3)
Polygamy (n = 7639)	
Monogamous	4603 (60.3)
Polygamous	1605 (21)
Don’t know	317 (4.2)
Not married	1114 (14.6)
Education level	
None	2020 (26.4)
Primary school	2910 (38.1)
Secondary school or higher	2717 (35.5)
Education level of partner (n = 7281)	
None	1508 (20.7)
Primary school	2238 (30.7)
Secondary school or higher	2903 (39.9)
Never married	632 (8.7)
Literacy (n = 7477)	
Literate	3790 (50.7)
Illiterate	3687 (49.3)
Wealth quintile	
1st (poorest)	1606 (21)
2nd	1585 (20.7)
3rd	1542 (20.2)
4th	1540 (20.1)
5th (wealthiest)	1374 (18)
Religion (n = 7555)	
Christian (catholic or protestant)	5015 (66.4)
Muslim	1890 (25)
Other/none	650 (8.6)
Location of home	
Urban	3472 (45.4)
Rural	4175 (54.6)
Own a method of transportation (bicycle, motorcycle or car) (n = 7635)	
Yes	3066 (40.2)
No	4569 (59.8)
HIV status (n = 3544)	
Positive	177 (5)
Negative	3367 (95)
Currently pregnant	
Yes	1061 (13.9)
No	6586 (86.1)
Parity	
1–2	3101 (40.5)
3–4	2117 (27.7)
5+	2429 (31.8)
Number of children <5 in the household	
0	472 (6.2)
1–2	4941 (64.6)
3+	2234 (29.2)
Current contraceptive use	
Modern method	1161 (15.2)
Traditional method	665 (8.7)
None	5821 (76.1)
Employment in the past 12 months (n = 7645)	
Yes	5657 (74)
No	1988 (26)
Antenatal care	
Visited a health facility in the past 12 months (n = 7637)	
Yes	5058 (66.2)
No	2579 (33.8)
Antenatal care provider (at least once) (n = 7582)	
Physician	1714 (22.6)
Nurse	4254 (56.1)
Auxiliary nurse	507 (6.7)
None	1107 (14.6)
Antenatal care location (n = 7489)	
Hospital	2975 (39.7)
Health centre	3366 (44.9)
Home	41 (0.6)
No ANC visits	1107 (14.8)
Timing of initial ANC visit (n = 7609)	
First trimester	2620 (34.4)
Second trimester	3489 (45.9)
Third trimester	393 (5.2)
No ANC visits	1107 (14.5)
Number of ANC visits during pregnancy (n = 7565)	
0	1107 (14.6)
1–2	580 (7.7)
3	1122 (14.8)
4+	4756 (62.9)
Delivery location (n = 7580)	
Health facility	4952 (65.3)
Home	2628 (34.7)
Malaria prophylaxis in pregnancy	
Have a bed net at home for sleeping (n = 7644)	
Yes	2580 (33.7)
No	5064 (66.3)
Slept under a bed net on the night prior to the survey	
Yes	1291 (16.9)
No	6357 (83.1)
Took any malaria prophylaxis medication (n = 7608)	
Yes	4686 (61.6)
No	2922 (38.4)
Malaria prophylaxis medication received (n = 7742)^b^	
Sulfadoxine–pyrimethamine (SP)	3321 (42.9)
Quinine	691 (8.9)
Artemether–lumefantrine	285 (3.7)
Amodiaquine	258 (3.3)
Chloroquine	123 (1.6)
Other medication	242 (3.1)
None/don’t know	2822 (36.5)
Number of SP doses received (n = 7492)	
0	4189 (55.9)
1	1262 (16.8)
2	1072 (14.3)
3+	969 (12.9)
Source of malaria prophylaxis medication (n = 3242)	
ANC clinic	3170 (97.8)
Other medical clinic	32 (1)
Other source	40 (1.2)

Data adjusted for weighting, clustering and stratification

^a^ Numbers in parenthesis show the denominator for each variable due to missing data

^b^ More than one response allowed

## Results

The 2011 DHS survey captured 7647 women with a reported pregnancy in the previous 5 years. The median age of this group was 27 years, the literacy rate was 50.7% and 1/3 had attended secondary school or higher (Table 1). There was a representation of women from across the country in urban and rural settings, and most women were married (68.6%), Christian (66.4%) and employed (74%). Access to ANC care was good with 85.4% reporting at least one visit, 62.9% reporting at least four visits and 45.9% had their initial visit during the second trimester. Nearly 60% had a parity of 3 or more.

Only 33.7% of women reported having a bed net at home, and 16.9% had slept under a net on the night prior to the survey. Of the 3303 women who took SP, 38.2% received 1 dose, 32.5% received 2 doses and 29.3% received 3 or more doses (Table 1). The subset of women who were pregnant at the time of survey (n = 1061) had similar findings, with 32% bed net ownership and 15% bed net usage. Nearly 2 in 3 women used medication for malaria prophylaxis during their most recent pregnancy and in this group, most took SP (67.5%).

### Bed net ownership

Table 2 shows the results of the multivariable analysis. In terms of bed net ownership, the univariate analysis showed an association with maternal primary school education (compared to no education), owning a method of transportation, the presence of children under age 5 in the home and measures of improved access to healthcare. Women who were not married were less likely to own a bed net. In the adjusted models, bed net ownership was associated with the presence of children under 5 in the home (aOR 2.3, 95% CI 1.7–3.1 for 3 or more children), more frequent ANC visits (aOR 1.6, 95% CI 1.3–1.9 for 4 or more visits), maternal literacy (aOR 1.2, 95% CI 1.02–1.5) and ownership of transportation (aOR 1.2, 95% CI 1.1–1.4). There was very little regional variation in bed net ownership.

**Table 2:** Multivariable logistic regression models to determine factors associated with use of malaria prophylaxis tools in pregnancy

Characteristic	Bed net ownership (n = 7235)	Bed net usage (n = 7239)	Use of any prophylaxis medication (n = 7226)
	Adjusted OR (95% CI)	Adjusted OR (95% CI)	Adjusted OR (95% CI)
Age			
Per 10 years	1 (0.9–1.1)	1.1 (0.9–1.2)	1.1 (1–1.2)
Education			
None	Ref	Ref	Ref
Primary	1.2 (1–1.4)	1.5 (1.1–2.1)	1.3 (1.05–1.6)
Secondary	0.9 (0.7–1.2)	1.1 (0.7–1.8)	1.5 (1.1–2.1)
Literacy			
No	Ref	Ref	Ref
Yes	1.2 (1.02–1.5)	1.4 (1.1–1.8)	1.4 (1.1–1.7)
Wealth quintile (lowest to highest)			
1st	Ref	Ref	Ref
2nd	1 (0.8–1.2)	1 (0.7–1.3)	1.1 (0.9–1.4)
3rd	0.9 (0.8–1.2)	0.9 (0.7–1.3)	1.1 (0.8–1.3)
4th	1 (0.7–1.2)	0.9 (0.6–1.2)	1.2 (1–1.6)
5th	1 (0.8–1.3)	1 (0.7–1.4)	1 (0.7–1.3)
Religion			
Muslim	Ref	Ref	Ref
Christian	0.9 (0.8–1.1)	1.2 (1–1.6)	0.8 (0.6–0.95)
Other/none	0.8 (0.7–1.1)	0.9 (0.7–1.4)	0.7 (0.5–0.98)
Parity			
1–2	Ref	Ref	Ref
3–4	1 (0.8–1.2)	1 (0.9–1.3)	1.2 (1.02–1.4)
5+	1 (0.8–1.3)	1 (0.8–1.3)	1 (0.8–1.2)
# Children under five at home			
0	Ref	Ref	Ref
1–2	1.9 (1.5–2.5)	2.3 (1.6–3.3)	1.1 (0.8–1.4)
3+	2.3 (1.7–3.1)	2 (1.3–3)	1.1 (0.8–1.4)
Relationship status			
Married	Ref	Ref	Ref
Living with partner	0.8 (0.7–1)	0.9 (0.7–1.1)	0.8 (0.7–0.97)
Widowed, divorced or separated	0.8 (0.6–1)	0.6 (0.4–0.8)	0.8 (0.6–1.1)
Never in union	0.8 (0.7–1)	0.9 (0.7–1.1)	0.7 (0.6–0.9)
Transportation			
No	Ref	Ref	Ref
Yes	1.2 (1.1–1.4)	1 (0.8–1.1)	1 (0.9–1.2)
Current contraception			
None	Ref	Ref	Ref
Traditional	1.2 (1.01–1.5)	1.2 (0.9–1.5)	1.1 (0.9–1.4)
Modern	1 (0.8–1.2)	1 (0.8–1.2)	1 (0.9–1.2)
Visited health facility in past year			
No	Ref	Ref	Ref
Yes	1.1 (1–1.3)	1.1 (0.9–1.3)	1.3 (1.1–1.5)
Total ANC visits			
0	Ref	Ref	Ref
1–2	1.4 (1.03–1.8)	2 (1.4–2.8)	10.3 (7.2–14.6)
3	1.3 (1.1–1.7)	1.8 (1.2–2.6)	14.6 (10.6–20.2)
4+	1.6 (1.3–1.9)	2 (1.4–2.8)	17.8 (13–24.5)
Delivery location			
Home	Ref	Ref	Ref
Facility	1 (0.8–1.2)	1.3 (1.1–1.7)	1 (0.8–1.2)

Multivariable logistic regression models were adjusted for weighting, clustering and stratification. All variables in the table were included in the model

*Ref* referent value, *SP* sulfadoxine–pyrimethamine, *OR* odds ratio

### Bed net usage

Factors in the unadjusted models associated with bed net usage (among those who owned a bed net) included older maternal age, higher educational level (woman and partner), literacy, higher wealth quintile, use of contraception and having children under age 5 in the home. Decreased bed net usage was associated with polygamy and owning a means of transportation. Regional variation was noted in bed net usage: women in more populated regions (such as urban Douala and Adamaoua) had higher usage than women in the more sparsely populated Extreme North region. After multivariable adjustment, usage was associated with a higher number of children under age 5 in the home (aOR 2, CI 1.3–3 for 3+ children), number of ANC visits (aOR 2, CI 1.4–2.8 for 1+ visit), maternal primary school education (aOR 1.5, CI 1.1–2.1), maternal literacy (aOR 1.4, CI 1.1–1.8) and having a facility delivery (aOR 1.3, CI 1.1–1.7). Women who were widowed, divorced or separated were less likely to use a bed net compared to women who were married (aOR 0.6, CI 0.4–0.8). Wealth quintile and maternal age did not predict any of the outcomes in the adjusted models. When the model was run with urban/rural residence replacing wealth index (two collinear variables), it was not associated with the outcome.

### Malaria prophylaxis medication use

In the univariate models for usage of any malaria prophylaxis medication during the most recent pregnancy, an association was seen with literacy, education, higher wealth index, urban residence, HIV-positive status, number of ANC visits and facility delivery. Polygamy, having an older partner, parity >5, ANC care at a health centre (compared to a hospital clinic) and later presentation for ANC care were negatively associated (data not shown). In the adjusted models, an independent association was seen for more ANC visits (aOR 17.8, 95% CI 13–24.5 for 4+ visits), maternal education (aOR 1.5, 95% CI 1.1–2.1 for secondary compared to none), maternal literacy (aOR 1.4, 95% CI 1.1–1.7), having visited a health facility in the past year (aOR 1.3, 95% CI 1.1–1.5) and higher parity (aOR 1.2, 95% CI 1.02–1.4 for 3–4 compared to 1–2).

Factors associated with the use of higher doses of SP (three separate outcomes) are displayed in Table 3. The factor most strongly associated with increased SP dosing was a higher number of ANC visits (aOR 25.4, CI 12.3–52.5 for the association of 4+ visits with 3+ SP doses). Maternal primary school education and having visited a health facility in the past year were associated with the receipt of at least one dose of SP and literacy was associated with the receipt of 1–2 doses of SP. Wealth quintile was not associated with the number of SP doses received. Parity greater than 2 had a negative association with receiving at least 2 doses of SP and a relationship status of “living with partner” or “never in union” was negatively associated with receipt of higher doses of SP compared to married women (aOR 0.6, 95% CI 0.5–0.8 for never in union).

**Table 3:** Multivariable logistic regression models to determine factors associated with the use of higher doses of sulfadoxine–pyrimethamine (SP) for malaria prophylaxis in pregnancy (n = 7121)

Characteristic	0 vs 1+ doses of SP	0–1 vs 2+ doses of SP	0–2 vs 3+ doses of SP
	Adjusted OR (95% CI)	Adjusted OR (95% CI)	Adjusted OR (95% CI)
Age			
Per 10 years	1 (0.9–1.2)	1.1 (0.9–1.2)	1.1 (0.9–1.3)
Education			
None	Ref	Ref	Ref
Primary	1.3 (1.04–1.7)	1.1 (0.9–1.3)	1.1 (0.8–1.5)
Secondary	1.3 (0.9–1.8)	1 (0.8–1.3)	1.4 (0.9–2)
Literacy			
No	Ref	Ref	Ref
Yes	1.3 (1.04–1.5)	1.3 (1.1–1.6)	1 (0.7–1.3)
Wealth quintile (lowest to highest)			
1st	Ref	Ref	Ref
2nd	1.1 (0.9–1.4)	1.1 (0.9–1.5)	0.8 (0.6–1.1)
3rd	1.1 (0.8–1.3)	1.2 (0.9–1.5)	0.9 (0.7–1.3)
4th	1 (0.8–1.4)	1.1 (0.8–1.5)	1 (0.7–1.4)
5th	0.8 (0.6–1.1)	1.2 (0.9–1.5)	1.2 (0.8–1.7)
Religion			
Muslim	Ref	Ref	Ref
Christian	0.8 (0.6–1.1)	0.9 (0.7–1.1)	0.9 (0.7–1.2)
Other/none	0.9 (0.7–1.3)	0.8 (0.6–1.1)	0.9 (0.6–1.4)
Parity			
1–2	Ref	Ref	Ref
3–4	0.9 (0.8–1.1)	0.8 (0.7–.98)	0.9 (0.7–1.1)
5+	0.8 (0.7–1)	0.7 (0.6–0.9)	0.8 (0.6–1.1)
# Children under five at home			
0	Ref	Ref	Ref
1–2	1 (0.8–1.3)	0.9 (0.7–1.2)	0.8 (0.6–1.1)
3+	1.1 (0.9–1.5)	1 (0.7–1.3)	0.9 (0.6–1.2)
Relationship status			
Married	Ref	Ref	Ref
Living with partner	0.6 (0.5–0.7)	0.7 (0.6–0.8)	0.7 (0.6–0.9)
Widowed, divorced or separated	0.8 (0.6–0.96)	0.9 (0.7–1.1)	1 (0.8–1.4)
Never in union	0.7 (0.5–0.8)	0.6 (0.5–0.8)	0.7 (0.5–0.9)
Transportation			
No	Ref	Ref	Ref
Yes	1 (0.8–1.1)	1 (0.9–1.2)	1 (0.9–1.2)
Current contraception			
None	Ref	Ref	Ref
Traditional	1.2 (0.9–1.4)	1 (0.8–1.2)	1.2 (0.9–1.5)
Modern	1 (0.8–1.2)	1 (0.8–1.2)	1 (0.7–1.3)
Visited health facility in past year			
No	Ref	Ref	Ref
Yes	1.2 (1.05–1.4)	1 (0.8–1.1)	0.9 (0.7–1.1)
Number of antenatal clinic visits			
0	Ref	Ref	Ref
1–2	19.4 (11–34.4)	10.2 (5.3–19.5)	3.5 (1.4–8.5)
3	30.5 (17.8–52.4)	24.8 (12.9–47.8)	18.9 (8.8–40.3)
4+	37.4 (22.1–63.2)	33.1 (17.8–61.8)	25.4 (12.3–52.5)
Delivery location			
Home	Ref	Ref	Ref
Facility	1 (0.9–1.3)	1 (0.8–1.2)	0.9 (0.7–1.2)

Multivariable logistic regression models adjusted for weighting, clustering and stratification. All of the variables in the table were included in the model

*Ref* referent level, *SP* sulfadoxine–pyrimethamine, *OR* odds ratio

**Fig. 1:**
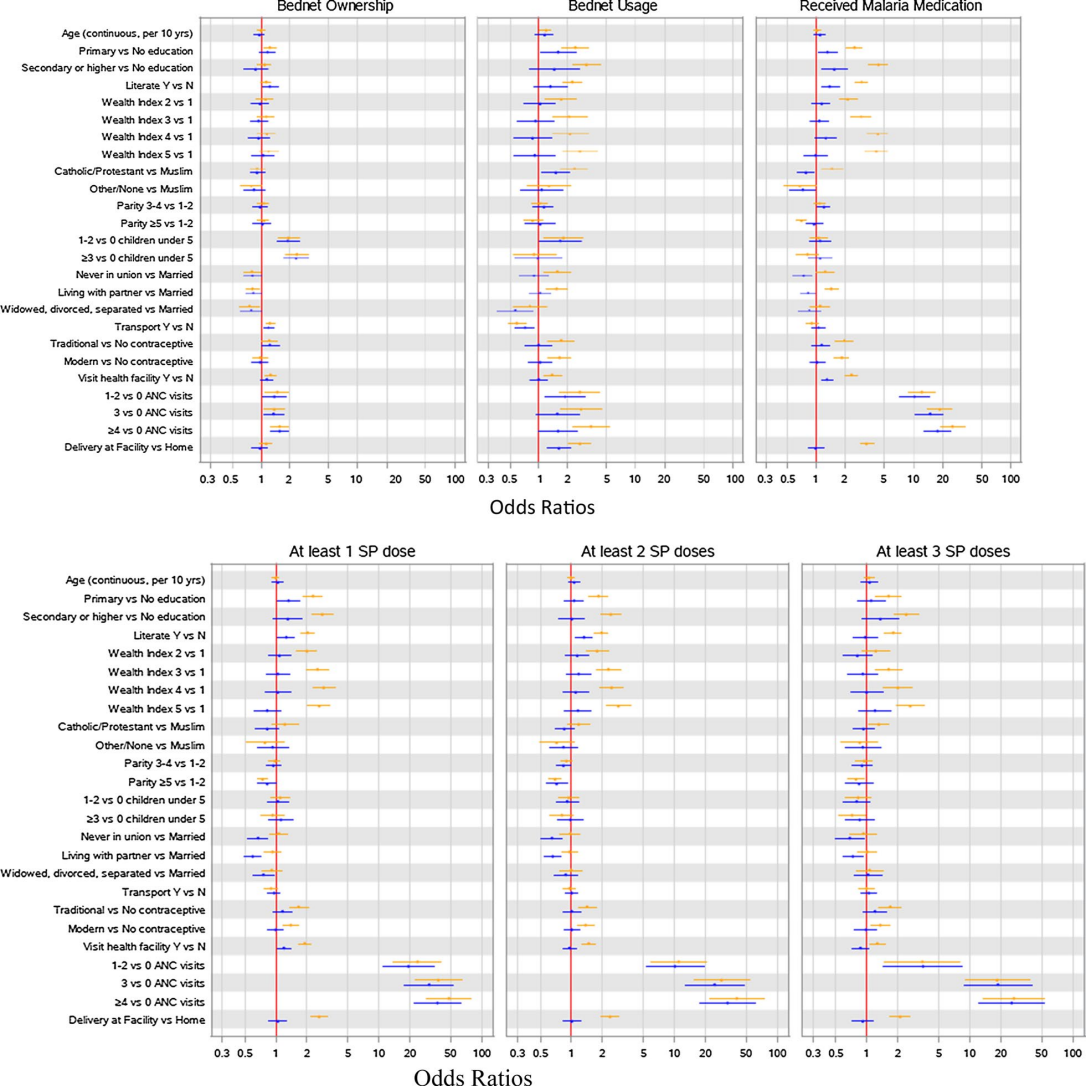
Odds ratios from univariate (*orange*) and multivariable (*blue*) logistic regression models (adjusted for weighting, clustering and stratification) by outcome

Figure 1 shows the differences in odds ratios and 95% CIs between the UV and MV models by outcome. It highlights the importance of the MV model in adjusting for covariates and the primacy of ANC care in terms of the medication-related outcomes.

## Discussion

This study of more than 7500 women with a recent pregnancy in Cameroon highlights the poor utilization of available tools for malaria prevention despite persistent and endemic disease rates. This finding is consistent with prior studies and particular characteristics were identified to be associated with intervention use at the individual, provider, system and community levels. This information is relevant to inform future prevention efforts.

### Bed net ownership

Only one in three women with a recent pregnancy owned a bed net. There was a wide range of bed net ownership (3–80%) documented by Singh et al. among pregnant women in sub-Saharan Africa [13]. The strongest predictors of net ownership in our study were maternal literacy, the number of young children in the house, and antenatal care. The association with maternal education is consistently seen, but the current analysis did not confirm the previous association with wealth index, parity or urban residence [13]. Also, bed net ownership rates were similar in all regions of the country. These positive findings point to equity in net ownership which may be attributed to public health campaigns that aim to disseminate bed nets widely at no cost [25]. The strong association with the presence of young children at home is likely due to patterns of prioritized net distribution for these households in the past [13, 23].

### Bed net usage

In this study, bed net usage rates were low at 16.9%. The rate of net usage during pregnancy was similarly low in Nigeria (26%) and 17% in sub-Saharan Africa overall [13, 14]. The clear discrepancy between net ownership and usage in pregnancy has been documented and some of the cited explanations include: discomfort, torn nets and a perception that nets are unnecessary [13, 26]. Maternal education, literacy, wealth, urban residence and older age have been variably associated with bed net usage in earlier studies but only maternal literacy and education were associated in this adjusted model [13, 27]. One explanation for this difference may be that public health messaging in support of bed nets in Cameroon has reached a broader population, including younger women, those with a lower socioeconomic status or women in rural areas but the major message is that usage rates are too low. This study, like others, supports the need for long-term investment in the education of girls.

### Use of prophylaxis medication

Only 6 in 10 women were prescribed medication for malaria prophylaxis during pregnancy and about one in four (27.2%) received the recommended 2 or more doses of SP. This is consistent with the recent World Malaria Report, where 57% of pregnant women in sub-Saharan Africa received SP, 43% received 2 doses and 17% received 3 or more doses [21]. Nonetheless, prophylaxis rates have improved significantly in sub-Saharan Africa (SP coverage was 19% in 2007) [14]. The strong association between the number of ANC visits and use of prophylactic medication is expected since these medications are most often administered from ANC clinic. There is an obvious clinical benefit to 2 or 3 doses of SP compared to single dose and longer time (>10 weeks) between doses has been associated with suboptimal outcomes [28]. Wealth was associated with access to and use of malaria prophylaxis medications in prior studies but was not seen in this study [21, 29]. Access to malaria prophylaxis among pregnant women may have become more equitable in Cameroon due to affordable essential drug schemes which lower costs for all.

### Strengths and weaknesses

The strengths of this study include its size and the survey methods involving weighted data from national household surveys designed to be representative. The comprehensive data collection allowed for exploration of several individual level factors as well as measures of healthcare access. Limitations of this study include the single country design, use of data from 2011 and self-reported measures. A few additional limitations inherent to the survey methods include recall bias (pregnancy up to 5 years before the survey) and social desirability bias (the desire to give the “correct” answer to the person administering the survey). Analysis based on region, ANC provider type or facility type was not performed because of collinearity. Most of the women queried about bed nets were not pregnant at the time of the survey but were of childbearing age with a recent pregnancy and this was used as a proxy for the behavior of women during pregnancy. Bed net usage was defined by the standard indicator of having slept under a net on the night prior to the survey. This may not be best indicator of actual usage although it is commonly used and it is the outcome for the “Roll Back Malaria Initiative”. Pregnancy outcomes related to malaria infection were not available. Finally, because of multiple comparisons, some of the associations noted may have occurred by chance alone.

## Conclusions

This study highlights important opportunities to optimize malaria prevention efforts in pregnancy in Cameroon. Insecticide-treated bed nets should be offered universally and use encouraged in antenatal clinic. Community-based bed net distribution should continue since households with access to a means of transportation were more likely to own a net and women living in more rural regions were less likely to sleep under nets. In order to reach the 2016 WHO malaria indicator of “the proportion of pregnant women who receive at least 3 or more doses of IPT in ANC clinic”, women should be encouraged to seek prenatal care early in pregnancy and providers should recognize every visit as an opportunity to discuss, recommend and offer malaria prevention tools. Since women with children under 5 years old were more likely to have and use a bed net, targeted efforts to educate primiparous women about malaria prevention in pregnancy may be useful. On a broader scale, education must remain a national priority in Cameroon given the relevance of maternal literacy and level of parental education as it impacts the use of malaria prevention tools.
